# The efficacy of applying the Interpersonal Effectiveness skills of dialectical behavior therapy into communication skills workshop for clinical nurses

**DOI:** 10.1016/j.heliyon.2023.e14066

**Published:** 2023-03-03

**Authors:** Shu-I Wu, Shen-Ing Liu, Yih-Jer Wu, Ling-Lang Huang, Thih-ju Liu, Kai-Liang Kao, Yu-Hsia Lee

**Affiliations:** a Department of Medicine, MacKay Medical College, New Taipei City, Taiwan; b Section of Psychiatry and Suicide Prevention Center, MacKay Memorial Hospital, Taipei, Taiwan; c Department of Cardiology, MacKay Memorial Hospital, Taipei, Taiwan; d Department of Nursing, Mackay Memorial Hospital, Taipei, Taiwan; e Department of Pediatrics, Far Eastern Memorial Hospital, New Taipei City, Taiwan

**Keywords:** Interpersonal effectiveness, Dialectical behavior therapy, Professional fulfillment, OSTE, Communication skills

## Abstract

**Background:**

We designed this open-pilot study to investigate the efficacy and feasibility of incorporating the Interpersonal Effectiveness skills from Dialectical Behavior Therapy (DBT-IE) into a 3-h clinical communication workshop for registered nurses.

**Method:**

A convenience sample of registered nurses were invited. The Professional Fulfillment Index, Perceived Stress Scale, Empathy Index, the Interpersonal Reactivity Index, and measures regarding quality of life, anxiety, depression, and insomnia were completed. A subgroup of participants received the Objective Structured Teaching Examinations (OSTE). Pre- and post-workshop assessments were conducted to identify the most empathetic or validated responses from case scenarios and to assess the self-rated levels of confidence regarding the capability to select the best answer. The satisfaction of the participants with respect to the workshop content, process, and the lecturer were also collected. Paired *t*-test was used for statistical analysis.

**Results:**

Among the 164 participants of the clinical communication workshop, 72 consented and their pre- and post-results were analyzed. Post-workshop assessment revealed significant improvement in professional fulfillment (p = 0.014), interpersonal coping ability (p = 0.038), and decrease in dysfunctional coping style (p < 0.001). The overall satisfaction score of participants was 4.68 (5-point Likert scale). In the subgroup that underwent pre- and post-workshop OSTE (n = 28), there was a significant improvement in total scores, pass rates, ratings from observational supervisors, simulated students, and simulated patients after the workshop (p < 0.001).

**Conclusion:**

Our results demonstrated the effectiveness, acceptance, and feasibility of incorporating the DBT-IE skills into a clinical medical communication workshop through a teaching style comprising of rigorous interactions and hands-on practices.

## 1 Introduction

The medical community is increasingly concerned about the importance of well-being in healthcare professionals (HCP), as HCP's wellbeing not only affects the HCP themselves, but also their coworkers and patients [3,44]. More than one-third of HCP in the United States experience exhaustion and burnout [40]. Studies have found that physical and mental stress, emotional exhaustion, or job dissatisfaction among HCP are related to clinician turnover rate in lifetime practice [19,46], reduced patient compliance [18], increased patient dissatisfaction [25], decreased nursing quality, poor patient outcomes [41], impaired patient safety [26], or even increased medical errors [31]. Notwithstanding, higher professional satisfaction among HCP was also significantly associated with higher overall patients' satisfaction to care [25] and better patient safety [24]. Hence, recent research on the well-being of HCP has not only emphasized distress or burnout, but also professional fulfillment, because this sense of meaningfulness and self-efficacy are potential intrinsic rewards that motivate medical practice [21,42,44].

Literature showed that interventions of organizational- or individual-levels, such as workflow changes, programs in quality or bureaucratic improvements, meditation, or mindfulness, may help reduce some HCP's burnout [2,32,46], while communication skills trainings may be more likely to improve HCP's satisfaction and decrease the intention to leave the job [32]. Linzer et al. described that those who received interventions on improving communication skills had greater improvements in professional fulfillment or career satisfaction (OR = 3.1, p = 0.04), and trends in burnout (OR = 3.0, p = 0.08), or intentions to leave the job (OR = 4.2, p = 0.06) than controls [32]. Good communication skills of empathizing and expressing the understanding towards patients' biopsychosocial contexts and goals of treatments was found to be associated with better clinical outcomes, professional satisfaction, and might help protect HCP from burnout [27,47,49]. Effective communication skills can also help increase patients' satisfaction, leading to improved treatment adherence and patient safety [10,34]. Nonetheless, previous studies have described that courses of communication skills trainings had little or no beneficial effects on emotional exhaustion or personal accomplishment among HCP involved in the care of cancer patients, although some improvement in general communication skills was noticed[35,36]. The main reason may be that contents of these courses focused on general medical communication skills, such as ways to build relationships, to structure clinical interviews, to gather and explain medical information, or to end the clinical interview. These general medical communication strategies may be insufficient to explore or validate patients' emotional or psychological issues, or to provide appropriate supports instead of overt reassurance. Whether enhancing HCP's communication skills to have the assertiveness to express their understanding of patients' feelings during clinical practice may be helpful in improving interpersonal coping strategies was also less studied [2,36]. Besides, ways of teaching or interacting with participants in the workshop might also have an impact. Research has shown that communication workshops with simulations of real-life clinical situations through role-plays, interactive practices, discussions, feedbacks, or self-debrief may be more useful in improving skills [5,22] and reducing burnout in HCP than traditional lecture-based teaching [2,14].

The Dialectical Behavior Therapy (DBT) is a well-designed, manual-based cognitive behavioral strategy originally developed to help people with poor interpersonal skills and/or those with emotional dysregulation under stressful conditions [29,30,43]. The standard DBT skills training for patients with emotional dysregulation encompass modules of Mindfulness training, Interpersonal Effectiveness, Emotional Regulation, and Distress Tolerance. The content of each module is covered over a period of 6 weeks (2.5 h/week, total of 15 h). Participants of DBT are expected to learn relevant behavioral skills through completing assignments, sharing experiences, interactive discussions, role plays, and by receiving corrective feedbacks on new behavioral skills they have actually practiced in interpersonal situations. The DBT-Interpersonal Effectiveness (DBT-IE) subtopic emphasizes on learning strategies to achieve interpersonal goals through assertiveness, behavioral reinforcement, and effective empathy or validation skills, that can be applied to self and others. Although supported by strong evidence in reducing emotional dysregulation, depression [11], anxiety, and improving mental health [16], DBT has been mainly applied to people with significant psychopathology in psychiatric settings. No study has investigated whether providing contents of DBT interpersonal skills that includes behavioral strategies in communication skills training programs can help improve HCP's well-being or satisfaction toward their profession, or lessen burnout or exhaustion. Moreover, there is a paucity of appropriate curricular or postgraduate trainings on patient-centered communication skills for clinical nurses [8,36,38,39]. Hence, we designed this open-pilot study with the hypothesis that a training incorporating the DBT-Interpersonal Effectiveness (DBT-IE) skills, which has a particular focus on the balance between accomplishing interpersonal goals and building alliances without losing self-respect, together with the interactive teaching styles of the DBT program, may help improve scores on measures of professional fulfillment, work exhaustion, and interpersonal coping strategies before and after the workshop among postgraduate clinical nurses.

## 2 Methods

### 2.1 Study participants and study procedures

This study is a part of a larger project that aimed to investigate the effects of applying the DBT- Interpersonal Effectiveness and Emotional Regulation skills into clinical medical communications. We collaborated with the supervisors responsible for implementing continuing education in the Department of Nursing at a large medical center in Northern Taiwan. With a pre- and post-test design, our inclusion criteria were full-time registered nurses, aged between 20–65 years, and those willing to participate in the study and the 3-h DBT-IE communication workshop that could earn them postgraduate continuing education credits. The exclusion criteria were those that refused to participate in the workshop, the study, those that were absent, or left the workshop. The advertisement of the DBT- IE communication workshop was displayed and disseminated to head nurses, nurse leaders, and clinical nurses from different sectors of the hospital through the internal electronic bulletin boards since mid- September 2020 (T0: the time of invitation). Those willing to participate could enroll voluntarily through the online register system. The registration ended at three weeks before the start of the workshop. A week later (T1: which was also two weeks before the DBT- IE workshop), enrollees were invited to sign the informed consent and received the baseline assessment. The date of the DBT- IE workshop was the 18th of November 2020 (T2). The participants’ satisfaction with the DBT- IE course, the contents of the workshop, and the interactive teaching style of the lecturer were assessed immediately after the completion of the workshop (T3). Professional fulfillment, burnout (including Work Exhaustion and Interpersonal Disengagement), perceived stress, the level of empathy, interpersonal reactivity and skills, and other pre- and post-workshop psychological assessments, included levels of anxiety, depression, insomnia, and quality of life, were measured two weeks before (baseline, T1) and after the workshop (T4: the 2nd of December 2020). The reason why these mental health status were measured as secondary outcomes was because they were related with HCP burnout and that DBT-IE and communication skills training might help improve mental health and enhance overall well-being [2,14]. A subgroup of junior nurses further received Objective Structured Teaching Examination (OSTE) assessments two weeks before (T1) and after (T4) the workshop. Comparison of performance in the OSTE was also used as an outcome for subgroup analysis. Another DBT- Emotional Regulation workshop for clinical nurses was held in May 2021. Herein we only report the pre- and post-results from the DBT- IE workshop in November 2020. The project has been approved by the Ethical Committee of the MacKay Memorial Hospital (IRB number: 20MMHIS092e).

## 3 Materials

### 3.1 Dialectical behavior therapy

The standard DBT skills training is a 48-week (total of 120 h) program. The Mandarin version of the DBT skills manual has been translated and utilized in several open-pilot and randomized-controlled studies by the Department of Psychiatry and Center for Suicide Prevention of the MacKay Memorial Hospital in Taiwan [9]. Since participants in our study were registered nurses without severe impairments in their daily function, the goal of our DBT- IE clinical communication skills workshop was to allow these nurses to learn and apply better interpersonal communication skills with DBT-components in their daily and clinical environment after a 3-h workshop. The original 15-h-long DBT- IE module was condensed into a 3-h long workshop. Condensation of the contents and processes of all workshops were designed by two consultant psychiatrists and three registered nurse supervisors with over 18 years of clinical experience. The consultant psychiatrist responsible for addressing these contents in the workshop not only had 10 years of experience in DBT training and clinical practice, as well as 5 years of experience as a DBT adherence coder, but was also an associate professor involved in teaching clinical medical communication to medical students for 4 years.

The DBT-IE skills addressed in the workshop are described in Table 1. We started by introducing case scenarios, and invite participants to share in small groups why they thought it was necessary to learn interpersonal communication skills, to apply them in clinical work, as well as to clarify their myths regarding interpersonal relationships. We then explained the theory, goals, and contents of the DBT-IE Objective, Relationship, and Self-Respect Effectiveness skills, and explained how to decide the priority during an interpersonal interaction. Much time was spent on explanations and practices of Positive Reinforcement strategy and Validation skills. We asked the participants to practice and role play on how to describe (D), express (E), be assertive (A), appear confident (A), be mindful and neglect attacks (M), plan for negotiation (N), and most importantly, to learn how to positively reinforce (R) [30] patients, their family, or persons they need to communicate with. Trainees were required to write down their own scripts for using the “DEAR MAN” skills when facing the case scenario and the clinical situations. Different levels of validation strategies were also taught. To validate others is not only to empathize “other person's feelings, wants, difficulties, and opinions,” [30] but also “to acknowledge” [30] or convey such acknowledgment through verbal and nonverbal cues with a nonjudgmental stance [29]. Participants then had the chance to write down their scripts and lines for applying these skills onto the case scenarios, and to share their opinions after role-play practice in pairs (Table 1). After submitting their scripts worksheets online, the lecturer went over their content, provided immediate feedback, and invited role plays. An essence of the communication workshop was the style to address these contents. We therefore particularly emphasized on the participants' immediate practice of skills through completing online worksheet, group discussions, role plays, and interactive feedbacks during the workshop.

**Table 1:** Summary of the content of clinical medical communication workshop applying the Interpersonal Effectiveness skills from Dialectical Behavior Therapy among clinical nurses.

Parts	*Contents*	*Techniques or hands-on practices*	*Duration (minutes)*
1.	Introduce case scenarios to motivate participants.	Video clips, discussion; invite opinion sharing.	3
2.	Why is it necessary to learn interpersonal skills and apply them in clinical work?	Interactive discussions and opinion sharing.	5
3.	Clarify the myths regarding interpersonal relationships from our participants.	Online-Google worksheet exercise for participants to select their most common myths regarding interpersonal interactions. Invite opinions and experience sharing on why they had such myths.	5
4.	Explain the theory and goals of DBT-Interpersonal Effectiveness skills (including Objective Effectiveness, Relationship Effectiveness, and Self-respect Effectiveness skills) and the key priorities.	Lecture and video clips on the theory and mnemonic chant. Use examples from case scenarios and invite participants to rank their priorities on Google worksheet. Discussion in pairs about priority setting.	15
5.	Particular explanation on ‘Positive Reinforcement skills’ from Objective Effectiveness skills.	Lecture, video clips, and QR-code scanning for online interactive quiz to assess whether the participants were able to understand and apply the idea of positive reinforcement immediately.	20
6.	Break		10
7.	Explain and practice ‘Mindfulness skills’ from Objective Effectiveness skills. Particularly focus on the nonjudgmental stance.	Lecture, video clips, and mindfulness listening practice. Invite discussion in pairs and opinion sharing in the class.	10
8.	Further explanations on ‘Validation’ Levels 1–5 from Relationship Effectiveness skills: Level 1: Listen and express interest; Level 2: Summarize and reflect; Level 3: Identify and articulate the unspoken emotions, thoughts, or behaviors; Level 4 and 5: Validate that the person's feelings or thoughts were understandable in the context of the person's past history or current situation without mistakenly reinforcing dysfunctional behaviors. Level 6: Empower the person and treat him/her as an equal partner.	Lecture, video clips, role-plays on Validation levels 1–5; explain the differences between validation and empathy; discussion in pairs; and opinion sharing with the class.	20
9.	To apply these Interpersonal Effectiveness skills to the case scenario mentioned at the beginning of the workshop.	Invite participants to choose from their priorities and write directly on Google sheet their scripts and lines for applying these Interpersonal Effectiveness skills to the case scenario. Invite practice and role-plays in pairs and opinion sharing with the class regarding what they wrote and practiced.	25
10.	Break		10
11.	Explain ‘Determining the intensity of making requests or rejections during interpersonal interactions’	Lecture, video clips, and hands-on practices using Google worksheet. Participants may decide on the level of intensity they preferred based on their feedback scorings on the Google worksheet.	20
12.	Analyze and review as to how the content from the script and the ‘determining intensity’ worksheets written online may be modified and improved.	’‘’ Google worksheets. Invite practice and role-play in pairs and opinion sharing in the class.	20
13.	Questions and answer session to address the issues brought up by the participants.	Summarize their questions, use Interpersonal Effectiveness worksheets to review the principles learned, and conduct role-plays.	20

aThe DBT-Interpersonal Effectiveness skills include the Objective Effectiveness skills, Relationship Effectiveness skills, and Self-respect Effectiveness skills. Trainees have to analyze their own beliefs and myths when encountering interpersonal situations in clinical settings, and learn to prioritize the main goal, whether it's Objective, Relationship, or Self-respect Effectiveness, they want to achieve in clinical situations.

### 3.2 Assessments

The following questionnaires were used to measure the changes in the perceived sense of self-fulfillment, burn out, perceived stress, depression, anxiety, insomnia, or quality of life before and after the 3-h workshop.

1. Professional Fulfillment Index (PFI) and PFI Burnout measures [44]: There are 16 items in the PFI, which are used to assess clinicians' sense of professional self-realization (professional fulfillment subscale) and job burnout (Work Exhaustion and Interpersonal Disengagement subscales). Good test-retest reliability (α = 0.86–0.92 for different subscales) and strong correlation between the PFI Burnout subscales and the Maslach Burnout Inventory (r ≥ 0.50) were reported. The Mandarin version of the PFI was translated and back-translated by different clinicians fluent in both English and Mandarin. The Cronbach α for internal consistency of the different subscales in the sample of Taiwanese clinical nurses were 0.92 for Professional Fulfillment, 0.80 for Work Exhaustion, and 0.92 for Interpersonal Disengagement.
2. Perceived Stress Scale (PSS) [12]: PSS is the most widely used psychological tool to assess the degree of stress in a person's life. PSS contains questions about the thoughts and feelings related to perceived stress during the last month. The internal reliability for the Mandarin version of PSS in the sample of Taiwanese clinical nurses was Cronbach α = 0.80.
3. Empathy Index (EI) [48]: The EI is a 10-item self-reported measure of empathy developed by a focus group of 5 experts in the field of medical education in Taiwan. A previous study among registered nurses in Taiwan demonstrated moderate construct and convergent validity when compared with the Interpersonal Reactivity Index (IRI) [48]. In the sample of Taiwanese nurses, the EI showed good internal consistency (Cronbach α = 0.96).
4. DBT Ways of Coping Checklist (DBT-WCCL) [37]: The DBT-WCCL was adapted from the “The Ways of Coping Checklist” [45]. Factor analysis showed that two subscales of the “coping ability” used DBT skills and “dysfunctional coping styles,” such as “blaming others.” The Cronbach α coefficient for internal reliability of the Mandarin DBT-WCCL in the sample of Taiwanese clinical nurses were 0.90 for the subscale of “coping ability,” and 0.93 for “dysfunctional coping styles”.
5. IRI: The IRI has a total of 28 questions to measure individual differences with respect to the level of interpersonal relationship. A previous study demonstrated good internal consistency and moderate convergent validity of IRI [15]. The Cronbach α coefficient for internal reliability of the Mandarin version of IRI were 0.70 for the subscale of “Empathic Concern,” 0.82 for “Perspective Taking,” and 0.62 for “Personal Distress” among Taiwanese clinical nurses.
6. Quality of Life Enjoyment and Satisfaction Questionnaire Short Form (Q-LES-Q SF) [20]: The Mandarin version of the Q-LES-Q SF has been shown to have good psychometric property in Taiwan [28]. The internal consistency of the Mandarin Q-LES-Q SF in the sample of Taiwanese clinical nurses was 0.95.
7. The 7-items Generalized Anxiety Disorder (GAD-7) screening questionnaire [1]: GAD-7 score ≥5 is indicative of the possibility of anxiety. The internal consistency for the Mandarin GAD-7 in the sample of Taiwanese clinical nurses was 0.93.
8. The 9-items Patient Health Questionnaire (PHQ-9) [33]: We used the Mandarin version of PHQ-9 to screen the participants for the presence of depression. Use of Mandarin PHQ-9 cutoff score ≥10 was associated with a high sensitivity (0.86) for the diagnosis of depression. The internal consistency of Mandarin PHQ-9 in the sample of Taiwanese clinical nurses was 0.92.
9. The Insomnia Severity Index (ISI) [23]: The ISI has 7 questions that explores sleep difficulties. The internal consistency of Mandarin ISI in the sample of clinical nurses was 0.92.
10. The Objective Structured Teaching Examination (OSTE) [4]: Since our participants were invited to participate in the 3-h clinical communication workshop to enhance their communication ability, not only in clinical situations but also with teaching new staff, we had invited a subgroup of nurses to be accredited as clinical teachers to take the OSTE before and after the workshop. The OSTE is derived from the Objective Structured Clinical Exercise, and is used to evaluate the teaching ability of clinical teachers. It conducts examination tasks through simulated teaching situations. Participants' performances are rated by observational supervisors, simulated patients/family, and simulated students.
11. Written quiz before and after the workshop: We administered pre- and post-workshop quiz with three multiple-choice questions for participants to select the most empathic responses from case scenarios. Self-reported levels of confidence on their capability to select the best answer for these multiple-choice questions before and after the workshop were also rated.
12. Participants' overall satisfaction (a 5- point Likert scale, with 5 being very satisfied) with the workshop, the contents, and the process, as well as to the lecturer were also enquired.

### 3.3 Statistical analysis

Demographic information such as age, sex, professional experience (years), and workplace were described. Chi-squared test or *t*-test were used to assess whether there were significant differences between those who completed the workshop, pre-, and post-assessments and those who did not complete. Paired *t*-test was used to compare whether there were changes in scores of professional fulfillment, work exhaustion, perceived stress, psychological states, insomnia, empathy, or interpersonal reactivity before (T1) and after (T4) the workshop. *P* values < 0.05 were considered indicative of statistical significance. Considering the possible mean differences and standard deviation of mean differences on measures we used, and at a power of 80% with a 2-sided α value of less than 0.05, the estimated sample size was to recruit at least 30 participants. The Bonferroni method was applied to correct for multiple testing.

## 4 Results

**Fig. 1:**
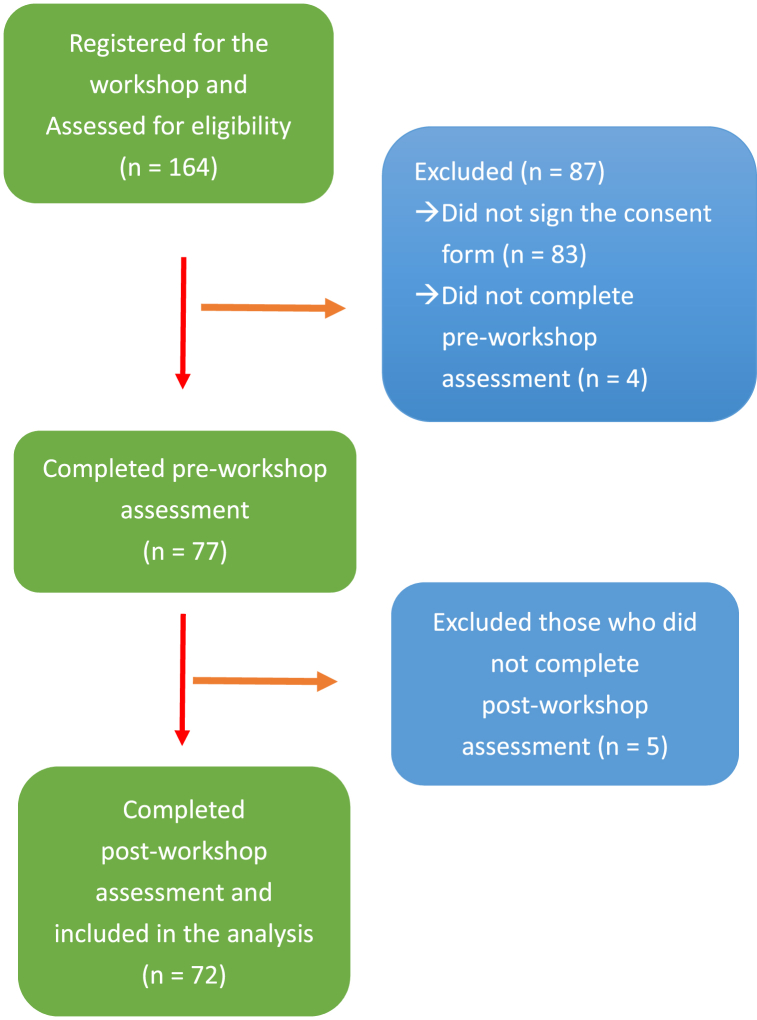
Study flow chart.

Among 164 enrollees of the clinical communication workshop, 81 (49.4%) nurses consented to participate in the study. Four nurses did not complete the pre-workshop assessment, and 5 nurses did not complete the post-workshop assessment. Thus, the pre- and post-workshop results of 72 participants were analyzed in this study (Fig. 1). These participants included registered nurses or leaders working in internal medicine or surgical wards, intensive or special care (e.g., hospice, hemodialysis, or oncology) units, or outpatient departments. Characteristics of participants that completed only the baseline assessment and those that completed both pre- and post-workshop assessment are compared in Table 2. No significant differences were noted between these two groups.

**Table 2:** Demographic data of participants (n = 77).

	Completed pre-test	Completed pre- and post-tests	Did not complete the post-test	Differences between those who completed posttest and those who did not (p-value)
	(n = 77)	(n = 72)	(n = 5)	
Basic information	n (%)			
Female	77 (100.0)	72 (100.0)	5 (100.0)	
Age groups				0.108
25–34	42 (54.5)	37 (51.4)	5 (100.0)	
35–44	23 (29.9)	23 (31.9)	0 (0)	
45–54	12 (15.6)	12 (16.7)	0 (0)	
Professional experience (years)				0.369
0–5	7 (9.1)	7 (9.7)	0 (0)	
6–10	33 (42.9)	29 (40.3)	4 (80.0)	
11–15	12 (15.6)	12 (16.7)	0 (0)	
16–20	10 (13.0)	9 (12.5)	1 (20.0)	
>20	15 (19.5)	15 (20.8)	0 (20.8)	
Work units				0.299
Wards	42 (54.5)	39 (54.2)	3 (60.0)	
Intensive care unit	19 (24.7)	19 (26.4)	0 (0)	
Special care unit (e.g., hospice, hemodialysis, or oncology)	12 (15.6)	10 (13.9)	2 (40.0)	
Outpatient department	4 (5.2)	4 (5.6)	0 (0)	

Table 3 shows that attending the DBT-IE clinical communication workshop was significantly associated with improvements in professional fulfillment (p = 0.014) and interpersonal skills assessed by the DBT-WCCL “coping ability of interpersonal skills” (p = 0.038), and a decrease in scores from the DBT-WCCL “dysfunctional coping style, such as blaming others” (p < 0.001). Nevertheless, the DBT-WCCL “coping ability of interpersonal skills” became insignificant after the Bonferroni correction (error rate of 0.025). All other psychological measures, including the GAD, PHQ, ISI, and Q-LESQ, did not show significant differences before and after the workshop, though a nonsignificant trend of decrease in depression was noted based on the PHQ-9 scores.

**Table 3:** Results of pre- and post-tests.

	Pre-test	Post-test	p-value
	Mean (SD)	Mean (SD)	
	(n = 72)	(n = 72)	
Professional Fulfillment Index			
Professional fulfillment	3.61 (0.61)	3.77 (0.65)	0.014
Work exhaustion	2.94 (0.69)	2.83 (0.71)	0.163
Interpersonal disengagement	2.30 (0.58)	2.26 (0.72)	0.616
Perceived stress scale	25.96 (6.39)	25.67 (5.62)	0.670
Empathy Index	37.56 (5.02)	37.54 (5.71)	0.965
Interpersonal Reactivity Index	91.94 (9.14)	91.93 (9.25)	0.989
Perspective taking	24.65 (3.21)	25.13 (4.10)	0.281
Empathic concern	25.11 (3.64)	25.20 (3.84)	0.807
Personal distress	21.86 (3.63)	21.49 (3.47)	0.348
DBT-WCCL^a^			
Interpersonal coping ability	2.21 (0.46)	2.37 (0.59)	0.038
Dysfunctional coping styles	1.96 (0.52)	1.63 (0.73)	<0.001
QLESQ	53.00 (8.12)	54.44 (8.89)	0.126
GAD	7.25 (4.29)	6.90 (4.51)	0.458
PHQ	8.60 (4.92)	7.57 (5.34)	0.065
ISI	9.20 (5.70)	8.95 (5.62)	0.652
OSTE total scores (n = 28)	47.50 (18.58)	75.45 (14.75)	<0.001
OSTE pass (n, %)	3 (10.3%)	20 (69%)	<0.001
OSTE Observational teachers rating	9.50 (3.72)	15.09 (2.95)	<0.001
OSTE Simulated student rating	6.50 (1.92)	8.07 (1.41)	0.002
OSTE Simulated patient rating	5.86 (1.98)	8.50 (1.17)	<0.001

Between-group differences assessed using the paired *t*-test.

a DBT-WCCL: Dialectical Behavior Therapy Ways of Coping Check List.

As for the subgroup of junior nurses that received the OSTE before and after the workshop (n = 28, Table 3), their total scores, pass rates, ratings from observational supervisors, simulated students, and simulated patients all showed significant increases after the workshop (p < 0.001). Improvements in nonjudgmental listening with open-ended questions, acknowledging and empathizing simulated patient's and student's emotions, using effective ways to describe, express, provide positive reinforcement, neglect attacks, and to provide appropriate information with less false reassurance or fact-only attitude (p < 0.001 for all) were noted compared to baseline.

With regards to pre- and post-workshop quiz containing case scenarios, although there was a trend of increase in correct rates after the workshop, no significant differences were observed compared to baseline. However, there was a significant increase in the participants' confidence in answering the same questions (p < 0.05 for all, Table 4). As for participants’ satisfaction scores on a 5-point Likert scale, an average of 4.68 was rated for the overall satisfaction, 4.72 for the contents and the process of the workshop, and 4.70 for the interactive teaching style of the lecturer.

**Table 4:** Results from pre- and postempathetic response tests.

	Pretest	Posttest	p-value
	n (%)	n (%)	
	(n = 72)	(n = 72)	
Question 1 (Correct answer)	38 (52.8%)	42 (58.3%)	0.503
Confidence (Mean, SD)	3.72 (0.88)	4.11 (0.83)	<0.001
Question 2 (Correct answer)	36 (50.0%)	45 (62.5%)	0.078
Confidence (Mean, SD)	3.71 (0.86)	4.04 (0.86)	0.001
Question 3 (Correct answer)	21 (29.2%)	26 (36.1%)	0.180
Confidence (Mean, SD)	3.78 (0.86)	4.10 (0.83)	<0.001

Between-group differences assessed using McNemar's test.

## 5 Discussion

To the best of our knowledge, this is the first study that applied the Interpersonal Effectiveness skills from the Dialectical Behavior Therapy (DBT-IE) to design a clinical postgraduate education workshop aimed to enhance interpersonal communication skills of registered nurses. Our results demonstrated that disseminating DBT-IE skills with DBT styles of interaction, role plays, and immediate generalization of newly acquired skills in class may be associated with an increase in the sense of professional fulfillment, and a decrease in their dysfunctional coping styles. We also observed a trend of decrease in the level of depression. The communication strategies and responses in simulated conditions also showed significant improvements in the post-test as demonstrated by better scores obtained from the OSTE than the pre-test. Levels of participants’ confidence in dealing with clinical case scenarios also improved.

In our study, we documented a significant improvement in professional fulfillment after the communication workshop. Increased self-reported levels of confidence on the capability to answer the multiple-choice written quiz after the workshop also demonstrated an increase in the level of self-efficacy. This finding is consistent with previous studies which showed improvement in self-efficacy after communication skills training programs [5,36], but no significant changes were found in the level of burnout [6]. The potential mechanisms by which DBT-IE workshop led to an increase in professional fulfillment is that the DBT-IE skills aim to facilitate trainees to achieve their goals in a clinical interpersonal situation without impairing their own self-respect, or their alliance with the patients, family, or team members. The course emphasizes on dispelling the myths that may interfere with Interpersonal Effectiveness since the very beginning, to motivate trainees to learn and apply these DBT-IE skills immediately during the workshop, and to generalize these skills to their clinical and daily life. Instead of directly teaching traditional communication skills, these learning processes have been designed to stimulate trainees to link these specific and constructive interpersonal coping skills to their daily life or work environment, to enhance their personal strengths, and to overcome weaknesses in communication in clinical medical settings. During the workshop, over half of the contents was spent on real-time practices using scripts writing on Google worksheets, role plays, discussions on case examples, and immediate feedbacks from peers and the lecturer to ensure that participants practiced these skills and were able to connect to the circumstances they were facing outside the workshop. These may be the reasons why the trainees felt more empowered in dealing with difficult interpersonal situations at work, or felt that they may contribute more professionally when facing clinical situations that require communication [17].

Our findings of the decrease in dysfunctional coping styles and the trend of improvement in interpersonal coping abilities (measured by DBT-WCCL) among trainees demonstrated that participants may have learned or noticed that they have better coping strategies and less emotional blame to self or others after the workshop. Different from previous studies that directly taught general clinical communication skills such as gathering information, breaking bad news, or sharing information with patients or family [7,35,36], in the DBT-IE workshop, we focused on more core interpersonal communication strategies, including how to balance between validating others and the assertiveness to accomplish one's goals after this communication (Table 1). Hence, the balance between “DBT-accept” (i.e., to validate patients or their family) and “DBT-change” (i.e., to encourage patients or family to make decisions or change their original attitude or behaviors regarding certain medical requests) was also a key focus. We believe that teaching and practicing these elements of DBT validation and reinforcement strategies might explain why participants showed improvement in their interpersonal coping styles. However, further studies are required to assess whether it affected professional fulfillment or whether these effects persist over time.

We found significantly higher OSTE total scores and ratings from observational supervisors or simulated patients and students after the DBT-IE workshop compared to baseline. This finding was in line with previous studies in which communication skills training programs for nurses were found to improve communication strategies [8,17,35,36]. Our results demonstrated ways to enhance empathic responses and provide appropriate information with clear and assertive attitude through the DBT-IE training. More importantly, the qualitative feedback obtained from simulated patients and students showed that trainees increased their uses of validation levels 1–5 (as described in Table 1) and positive reinforcement strategies. They felt that these skills were helpful in encouraging people express their emotions, anxiety, or difficulties in adapting the interpersonal situation. Such factors may explain the significantly higher pass rates and satisfaction ratings from observational supervisors and simulated students and patients.

Similar to previous research, although our communication workshop enhanced professional fulfillment and interpersonal skills among clinical nurses, there was no significant change in indices related to burnout, such as work exhaustion or interpersonal disengagement, anxiety, or quality of life [6,35,36]. This may be attributable to the fact that despite the substantial training contents of the workshop, only a single exposure of 3 h was used in consideration of clinical work arrangements. Prolonging the practice time in this single exposure or conducting a follow-up training may potentially improve the familiarity of the trainees with the relevant skills. Likewise, owing to the learning curve, it takes time to be well-acquainted to the skills taught and to apply them in the workplace. Follow-up assessments may be required to examine whether there were possible effects that may help decrease burnout [6]. Last but not the least, some other organizational factors may also affect burnout, such as excessive workload, time pressure, high job demands, low rewards, low job autonomy, or negative team atmosphere [13]. These burnout-related factors were not addressed in this workshop.

### 5.1 Strengths and limitations

Strengths of this study were that DBT-IE skills training is a manual-based cognitive behavioral technique that covers various aspects of interpersonal skills required in the modern medical setting. Our adherence to the original DBT's skills training, its interactive teaching style, and the delivery process emphasizing hands-on practices, group discussions and sharing, as well as immediate feedback on worksheets and role-play practices, might further enhance the efficacy of learning. The lecturer responsible for organizing and delivering the workshop was a clinician who was not only well-trained in DBT, but was also an expert in the field of mental health and in teaching communication skills. The use of multidimensional assessments including subjective, objective, and OSTE ratings were also a distinct advantage.

The key limitation of this study was that the post-test was administered 2 weeks after the workshop, and only changes within a short observation period were assessed. Follow-up assessments are still required to explore the long-term effects of this one-time workshop, the persistence of the effect over time, and whether any consolidation sessions would help enhance the communication skills or further improve interpersonal relationships in clinical settings. Second, although multidimensional assessments were performed, only a small subgroup received the OSTE. Further studies are required to assess the proficiency of adapting the skills taught at the workshop in real-world setting, and whether and how the knowledge gained transforms into specific changes in coping behaviors in clinical settings. Moreover, whether these behavior changes can help improve nurse or patient outcomes needs to be assessed. Third, those who consented to participate may have had greater motivation, readiness, or capability to change, or may have already had better interpersonal coping styles compared to those who did not consent to participate. Besides, we were only able to conduct a pre- and post-pilot study and not a randomized controlled trial. Without a comparison group, the results might have uncontrolled allocation or confounding biases. Fourth, the original PSS and the DBT-WCCL questionnaires were designed to investigate perceived stress or interpersonal coping styles in the past month. However, in our study, participants were administered pre- and post-tests two weeks before and within two weeks after the workshop date. Although we asked the participants to answer the post-test questions based on their conditions after the workshop, there might still be some deviations from the original design of these assessment tools. Finally, our participants for the workshop exclusively comprised of clinical nurses in Taiwan. Whether similar workshops may have similar effects on personnel in other medical specialties, or whether other non-workshop method may be superior or inferior to the current design requires further investigations. Further large-scale randomized-controlled trials with more objective evaluations and longer follow-up period are warranted to overcome these limitations.

## 6 Conclusion

Our results demonstrated improved professional fulfillment, interpersonal coping styles, and self-competencies after a short, 3-h workshop that focused on applying the DBT-IE skills onto clinical medical communication through a teaching style consisting of rigorous interactions and hands-on practices. The effectiveness, acceptability, and feasibility of the approach were also supported by participants’ satisfaction. Further studies with randomized-controlled design involving personnel in other medical specialties and with follow-up assessment are still warranted.

## Author contributions

Authors Shu-I Wu, Thih-ju Liu, Kai-Liang Kao, Yu-Hsia Lee designed the study and wrote the protocol.

Authors Shu-I Wu and Kai-Liang Kao undertook the statistical analysis.

All authors contributed to the writing and have approved the final manuscript.

## Conflict of interest

All the authors have no nonfinancial interests that may be relevant to the submitted work.
